# P-423. Cefazolin Versus Alternative Antibiotics for Preoperative Prophylaxis Among Children with a Reported Penicillin Allergy: Effect on Surgical Site Infection and New Drug Allergy

**DOI:** 10.1093/ofid/ofaf695.639

**Published:** 2026-01-11

**Authors:** Tracy N Zembles, Caroline Frahm, Cheryl Singer, Evelyn Kuhn, Richard Berens, Michael McCormick, Michelle L Mitchell

**Affiliations:** Children's Wisconsin, Milwaukee, WI; Children's Wisconsin, Milwaukee, WI; Children's Wisconsin, Milwaukee, WI; Children's Wisconsin, Milwaukee, WI; Medical College of Wissconsin, Milwaukee, Wisconsin; Medical College of Wissconsin, Milwaukee, Wisconsin; Medical College of Wisconsin, Milwaukee, Wisconsin

## Abstract

**Background:**

Cefazolin is the antibiotic of choice for prevention of infection for many pediatric surgical procedures. In 2018, we began utilizing cefazolin for pre-operative prophylaxis when indicated for all patients, including those with a reported penicillin (PCN) allergy. We made this change in practice because cefazolin does not share a side chain with any other beta-lactam, therefore risk of cross-reactivity in patients with a reported PCN allergy is expected to be no different than the general population.

**Figure:**
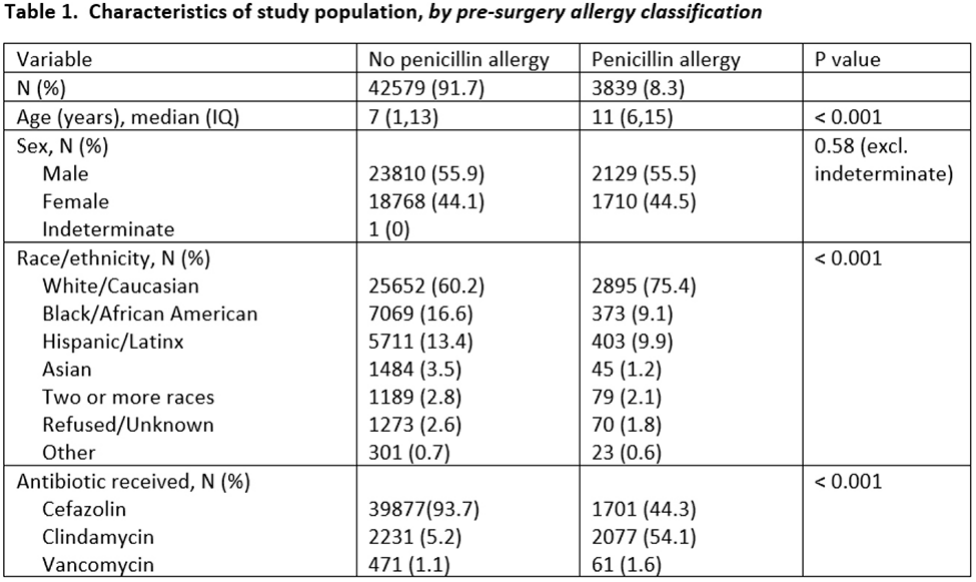

**Figure:**
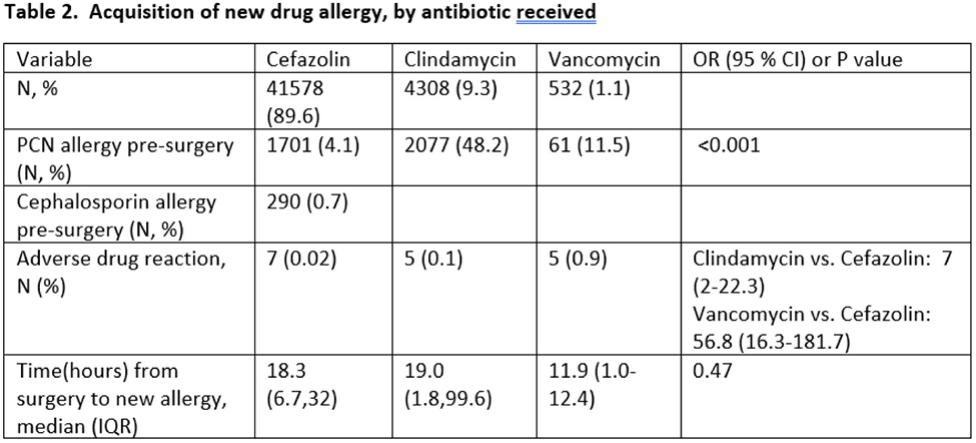

**Methods:**

We compared development of a surgical site infection (SSI) or acquisition of a new drug allergy among children (0-18 years of age) who received cefazolin, vancomycin, or clindamycin for an orthopedic, neurologic, cardiac, or plastic surgical procedure where cefazolin was the drug of choice over a 10 year time period (2013-2022). Outcomes were evaluated as a whole and stratified by pre-surgery allergy. SSIs were limited to procedures reported to the National Health Safety Network.

**Results:**

A total of 46,418 patients were included; 8.3% reported a PCN allergy (n=3,839) pre-surgery. Among those, 44.3% received cefazolin, while the remainder received clindamycin or vancomycin. Most patients (93.7%) without a PCN allergy received cefazolin. Of the total who received cefazolin (n=41,578), regardless of PCN allergy status, 7 (0.02%) experienced an adverse drug reaction. Five reactions were mild (grade 1) and 2 were moderate (grade 2). All seven occurred in patients who did not previously report a PCN allergy. Compared to cefazolin, patients were 7 times more likely (95% CI 2-22.3) to develop a new drug allergy to clindamycin and 56.8 times more likely (95% CI 16.3-181.7) to experience a drug reaction to vancomycin. There were no differences in SSI by type of antibiotic, type of surgery, or presence of PCN allergy.

**Conclusion:**

Drug reactions to cefazolin were rare. PCN allergy did not predict reaction to cefazolin. Cefazolin is safe to use in children, regardless of PCN allergy status at the time of administration.

**Disclosures:**

All Authors: No reported disclosures

